# A Secure Region-Based Geographic Routing Protocol (SRBGR) for Wireless Sensor Networks

**DOI:** 10.1371/journal.pone.0170273

**Published:** 2017-01-25

**Authors:** Ali Idarous Adnan, Zurina Mohd Hanapi, Mohamed Othman, Zuriati Ahmad Zukarnain

**Affiliations:** Faculty of Computer Science and Information Technology, Department of Communication Technology and Networks, Universiti Putra Malaysia, UPM 43300, Selangor, Malaysia; West Virginia University, UNITED STATES

## Abstract

Due to the lack of dependency for routing initiation and an inadequate allocated sextant on responding messages, the secure geographic routing protocols for Wireless Sensor Networks (WSNs) have attracted considerable attention. However, the existing protocols are more likely to drop packets when legitimate nodes fail to respond to the routing initiation messages while attackers in the allocated sextant manage to respond. Furthermore, these protocols are designed with inefficient collection window and inadequate verification criteria which may lead to a high number of attacker selections. To prevent the failure to find an appropriate relay node and undesirable packet retransmission, this paper presents Secure Region-Based Geographic Routing Protocol (SRBGR) to increase the probability of selecting the appropriate relay node. By extending the allocated sextant and applying different message contention priorities more legitimate nodes can be admitted in the routing process. Moreover, the paper also proposed the bound collection window for a sufficient collection time and verification cost for both attacker identification and isolation. Extensive simulation experiments have been performed to evaluate the performance of the proposed protocol in comparison with other existing protocols. The results demonstrate that SRBGR increases network performance in terms of the packet delivery ratio and isolates attacks such as Sybil and Black hole.

## Introduction

Rapid technological advancement of wireless communication devices and microprocessors have made wireless sensor networks (WSNs) technically and economically possible to be widely used in many real time monitoring applications related to both military and civilian [1, 2]. These applications could be used to gather physical information about the enemy movements on the battlefields or collecting sensitive information about the condition of patients in the medical fields.

A unique feature of these networks is their ability to deploy sensor nodes in a large number in an unattended fashion for a defined long period of time. The nodes, however, have a limited transmission range, which requires each node to communicate with its neighboring nodes to form a network for packets forwarding tasks. To accomplish these tasks sensor nodes also have to work under the influence of special configured routing protocol. Routing protocols are responsible to ensure that data packets are transmitted from a target region through multiple relay nodes to a destination without being dropped or/and compromised. Hence, it’s imperative important to secure the routing protocols against various routing attacks, especially when WSNs are used to transmit sensitive data packets to and/or from unprotected environments. This has become more vital since sensor nodes rely on wireless communication which is known to be vulnerable to attacks due to broadcast nature of the communication medium [2]. Together with security, issues such as coverage area [3, 4], network lifetime and energy efficiency [5, 6] as well as efficient routing [7, 8] have dominated the research activities for both Ad hoc and WSN.

Geographic routing (GR) protocol is an efficient and attractive approach for sensor network since no end-to-end route is established before data transmission. It also allows each node to keep the local one-hop connectivity that leads to network scalability [9]. GR forwards data packets as follows: a source node with packets wishes to send, broadcasts request-to-send (RTS) packet to its neighbors to initiate communication and the only neighbors within a restricted allocated sextant (forwarding area) close to the destination are eligible to reply with clear-to-send (CTS) packet to form a set of candidate forwarding nodes. Based on a certain selection criteria, the source node then selects a single node as a succeeding relay by complete reactive approach in which data packets are transferred to. Besides, the previous basic description of selecting a relay node of GR approach considers unusual situations. For example, in the presence of multiple attackers in the restricted allocated sextant, one or more attackers may become the first to reply with CTS, and when it is selected to become a next relay node, it may simply drop the data packets transferred to it or perform malicious activities on the data.

To prevent this kind of attacks, to date several secure routing protocols have been proposed [10–15], [9, 16–20]. Some of these protocols [10–12] make use of basic cryptographic tools such as primitive symmetric and/or asymmetric keys to secure routing process in WSN/MANET and safeguard transmitted data packets. However, such tools are known to be computationally expensive and incapable of meeting the resource-constrained property of the sensor networks [21–23]. These tools also are very effective in mitigating external attacks and perform poorly when internal attacks are of concern [24, 25]. Other protocols [13–15] are used to build a secure networking environment by allowing some nodes to monitor neighbours’ traffic behaviours and estimate trust values of each neighbouring node. Nonetheless, putting the nodes into monitoring mode to observe neighbours’ behaviours consumes a considerable high amount of nodes’ energy as well as more memory space [26]. Rather than relying on infrastructure such as special anchor nodes or/and distributed location verification algorithms, several other protocols [9, 16, 17] make use of a characteristic of wireless medium in the physical layer to provide some form of information verification of the nodes within a coverage area. These protocols utilized an available Received Signal Strength (RSS) of the node to offer security services to mitigate routing attacks in WSNs. However, the verification process in used of the nodes’ locations induces more cost on computations. Several other geographic protocols [18–20] have also been proposed to address the issue of security and resource limitations in sensor networks. These protocols have used extra forwarding logics and/or enhancing packet transmission principles to establish robust secure routing processes. Among these, Dynamic Window Secure Implicit Geographic Forwarding (DWSIGF) protocol [20] is the most recently proposed secure routing approach that guarantees the safety of the selection of next relay node in a given restricted (allocated) sextant to route data packets when attackers are in a communication link. The protocol uses a dynamic collection window time to collect a number of candidate nodes within that area in order to find an appropriate forwarding node (relay node) to relay packets to the destination.

However, when multiple adversaries are in the restricted sextant, the sextant as well as independency of message response for routing initiation mechanism may provide inadequate responses of candidate nodes from which to select a legitimate or an appropriate relay node. Moreover, when the attacker is selected from the sextant and drops packets, the source node needs to reinitialize communication through retransmission of control packets (ORTS). This may not guarantee the neighbors to successfully receive the control/data packets again next time due to the error-prone of wireless links which may lead to poor network performance. In addition, a dynamic collection window used provides insufficient collection time to collect enough nodes. Also, simultaneous verification is used to validate duplicate location of the nodes in order to avoid Sybil attack. However, in priority selection criteria, the attacker is still selected as a relay node.

The remainder of the paper is organized as follows: Section2 addresses related works on security techniques for routing protocols in WSNs and Sect.3 describes the proposed protocol. Sect. presents performance evaluations, and conclusion and suggestion of future work in Sect.5.

## Related Works

Many schemes have been proposed to provide defense against routing attacks for geographic routing in WSNs. This section provides related works on some of security techniques that have been proposed to address the issue. The techniques are described by highlighting mode of operations, strengths and weaknesses.

In [27] a trust-based defending model against multiple routing attacks is proposed for WSNs. Using Dirichlet distribution function, the model computes the trust value of each node based on the data packets’ acknowledgments instead of monitoring mode which demands more nodes’ communication resources. The model uses a combination of trust, the geographic position as well as energy aware metrics to select an appropriate node for routing. Its also employs dynamic trust policies to estimate the initial trust value of each node based on the feedbacks from three neighbouring nodes. Moreover, it then introduces an incentive mechanism to encourage more cooperative behaviours of the nodes for forwarding task. The model maintains better packet delivery ratio and improves network lifetime in contrary to the existing protocols. However, the employment of dynamic trust and incentive policies increases network communication overheads.

In [28], a secure position-based geographic forwarding algorithm (SGF) is proposed that incorporates security keys such as Hashed Message Authentication Code (MAC) [29] and the Timed Efficient Stream Loss-tolerant Authentication (TESLA) [30] with Instant Key disclosure (TIK) protocol to provides both sender and neighbour authentication which ensures message transmission integrity. It also integrates with a secure grid location service (SGLS) to facilitate any receiver to validate the correctness of the location of transmitted message. The location service SGLS provides extra security measures to the original GLS [31], by combining secure location querying and secure HELLO message exchanges. This measure prevent message spoofing, packet dropping as well as falsified message injection and reply attacks. However, the uses of heave authentication keys increase computation cost as well as message overheads.

In [32] a novel detection scheme for geographic routing protocol (DWGRP) is proposed to detect a wormhole attack. The scheme improves the pairwise key pre-distribution based on the beacon packets to effectively detect malicious nodes before the packet is received by a destination. Each node builds a neighbouring table to store neighbours’ identities and their initial-generated private keys based on the received beacon message. The table is updated when the nodes receive a new beacon from its neighbours. In order to build a trust path to send packet to the destination, source node creates a shared secure communicating key with its neighbours. This can be done by ensuring that one of the selected neighbours’ private keys comes from the same matrix with the source’s own private key, otherwise, the selected neighbour is eliminated from the neighbouring table. Even if the malicious node manages to breach the communicating shared key, it may fail the certification process conducted at the destination. The scheme maintains superior performance in detecting malicious nodes with low detection rate compared to other existing schemes. However, since sensor nodes are resource constraint devices, the generation of public/private keys demand a significant amount of computation and communication power, which quickly drains the sensor energy and causes a sudden death.

In [18], a Simple self-protected Beacon-less Geographic (SBGR) protocol is proposed to provide security for a transmitted packets to a destination. The SBGR enhances the basis of packet forwarding logic to provide defence against routing attacks. The forwarding logic is designed to prevent an attacker from terminating the communication when the suspicious data traffic is detected. The protocol addresses two types of attacks namely Sybil and Sink hole [33]. To prevent Sybil attacks, the SBGR used to flood a constrained NOTIFY packet to ensure that data are forwarded towards the destination when the position of replied node is suspected to be forged. In case of Sink hole, node needs to verify its position if it’s closer to the destination than other nodes that immediately replied with data packets. Having verified that, its position is nearer to the destination than to the rest of the neighbours, the node will forward packets towards destination while ignoring the replies of the other neighbours. In this way, the protocol manages to isolate the Sink hole attacks. However, the protocol incurs high communication overheads due to the flood of data packets when the attacker is detected.

In [19] the authors proposed a family of secure routing protocols (SIGF) that are designed to provide a flexibility to select a required protocol when a certain level of security threat is detected. In general, SIGF follow routing principles of Implicit Geographic Forwarding (IGF) [34] that is a nondeterministic Network/ MAC hybrid routing protocol that is completely stateless and based on 802.11 DCF MAC protocol. IGF handles network dynamics easily and basically restricts the effects of malicious node to a local region. Since the forwarding decision to select a single node is made as late as possible when a message is ready to be transmitted over the air. However, the protocol is susceptible to a simple attacker that rushes to be selected as relay node. In contrary, the SIGF extends IGF and populate the gap between pure statelessness and traditional shared-state security by developing three secure protocols namely SIGF0, SIGF1 and SIGF2. The SIGF0 provides defence against routing attacks by keeping no routing states of the nodes. In the SIGF1, the protocol uses local information and forwarding history to derive the reputation of each node and protect the protocol against insider attacks. The SIGF2 uses traditional cryptographic and sequencing mechanism for authentication as well as guarantees a strong security. However, the protocol is still susceptible to routing attacks caused by unresolved attacker when completely stateless approach is used [20].

In [20] DWSIGF protocol was proposed to reduce the attacker selection in the selection process of relay node within a given restricted allocated sextant. The protocol uses dynamic collection window time to select a single relay node to forward packets to the destination. The window is open dynamically to collect candidate nodes before applying selection criteria to select a single relay node. The single relay node is then picked either by priority or random selection criteria. In priority selection, any node that exhibits good residual energy and is near to the destination is selected to send packets further to the destination after simultaneous verification process to validate duplicate nodes’ locations. However, in random selection, a single relay node is picked from the set of candidate nodes. DWSIGF promises a good security defence against routing attacks without inserting any security mechanism in the routing protocol. However, the protocol lacks dependency on message responses for routing initiation as well as inadequate restricted allocated sextant to collect enough messages in order to select a single legitimate relay node to send packets to the destination. Independence and insufficient number of message responses from restricted allocated sextant may also create a retransmission of routing initiation (ORTS) packet when the attacker is selected and drop the data packets in which ultimately degrades network performance. It also provides insufficient collection window time to collect candidate nodes while the simultaneous verification process used in priority selection criteria still allows attacker to be selected as a relay node.

In order to address the aforementioned problems, a SRBGR protocol is proposed that it extends restricted allocated sextant (forwarding area) to a different area within the coverage of the source node so as to increase the number of CTS responders to participate in communication process when multiple attackers are in communication link. It then gives different contention priorities to the responders (i.e. CTS packet coming from each area- allocated restricted sextant and extended area) in order to improve the selection of CTS responders (legitimate nodes) coming from extended area. This may also reduce the retransmission of routing initiation packets. The proposed SRBGR also uses bound dynamic window to provide sufficient collection time for neighboring nodes to respond with CTS packets. It also uses the verification cost to validate node’s location in order to identify and isolate the attacker during priority selection criteria.

## Dynamic Window Secure Implicit Geographic Forwading (DWSIGF)

**Fig 1:**
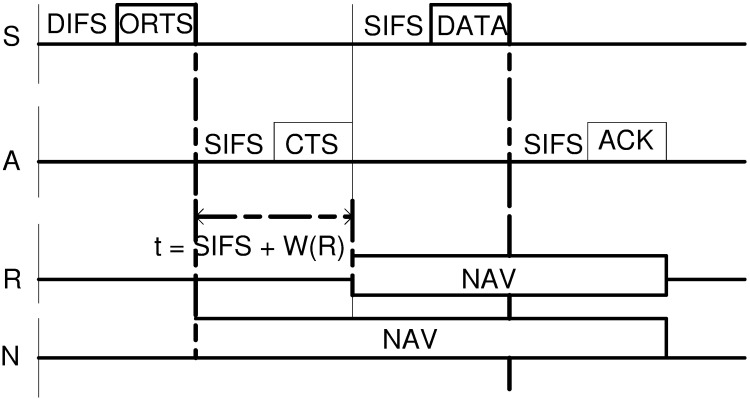
802.11 DFC Handshake Mechanism [34].

This section describe Dynamic Window Implicit Geographic Forwarding protocol. The routing principle of this protocol is based on the integration of Network/MAC Protocol of 80.2.11 DCF [34] as shown in Fig 1.

The mechanism begins when a Sender node S has a packet to send and its carrier senses a channel which is not in used for the duration of DIFS time while its Network Allocation Vector (NAV) timer is zero. If the unused channel is found, then Sender S broadcasts ORTS packet to neighboring nodes within the communication range of S.

This packet consists of the location of Sender S and Destination D. Neighboring nodes located in the forwarding area (60 sextant—*FrwdAreaSector*) are qualified to forward CTS packet. These nodes are called candidate (forwarding) nodes and usually set their CTS Response time inversely proportional to the weighted sum of their distance from the Sender S, residual energy and perpendicular distance from the Sender to the Destination. Other non-forwarding nodes suppress their CTS responses by setting their NAV timer in accordance with 802.11 semantic to mitigating interference. On the other hand, the candidate node with a shortest CTS Response time (i.e. which expires first) replies with CTS packet. The Sender node then collects a number of CTS responses within a dynamic collection window period. The window period creates a possible time shift in the protocol semantics [20, 35].

The collected CTSs are then immediately sampled to select the best next relay node (next hop) based on distance merit in two forwarding criteria namely; random or priority. In priority, any node shows the best progressive distance towards the destination is selected. However, in random any node is selected as long as it has managed to reply with a CTS packet which signifies that it has a positive distance toward the destination. After a single node (i.e. a relay node) has been selected, the Sender S continues with a communication in accordance with 802.11 DCF semantics (*ORTS* ⇒ *CTS* ⇒ *DATA* ⇒ *ACK*) utilizing an isolated link with the receiver [20, 35].

DWSIGF promises secured environment in selecting a relay node, however, the protocol lacks dependency on message responses for routing initiation as well as inadequate forwarding sextant to collect sufficient competing nodes in order to select a single relay node to send data to the destination. Furthermore, it provides insufficient collection window time to collect candidate nodes while the simultaneous verification process used in priority selection criteria still allows attacker to be selected as a relay node.

## Proposed Secure Region-Based Geographic routing Protocol (SRBGR)

### System, Network and Attack Model

**Fig 2:**
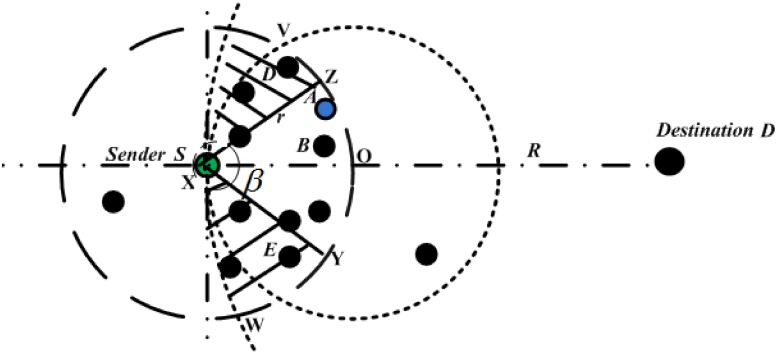
Extended Forwarding Area in SRBGR.

**Fig 3:**
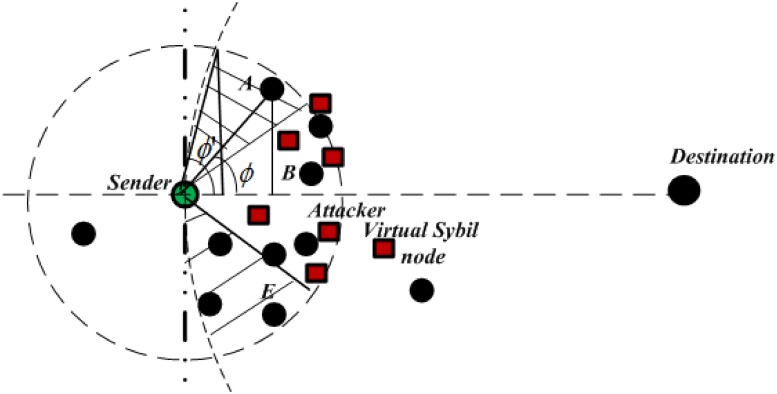
Increase Attackers in the Forwarding Area.

In the proposed protocol, the greedy forwarding area is divided into two sub-areas: restricted allocated sextant and secure extended area. The restricted allocated sextant is similar to the forwarding area used by previous protocols [19, 20, 34]. This area is within 60 degrees sextant centered that includes a 30 degree on both sides of the direct line connecting the sender and the destination. On the other hand, unlike the previous protocols, in the proposed protocol, the restricted allocated sextant is extended to include another region named as *a secure extended area* which is laid between the overlapping area created based on the radius of transmission range of the sender and the distance from the destination to the sender subtracting the area of restricted allocated sextant as shown in Fig 2 as a shaded area. The area is assumed to be secured since it is immune (insusceptible) with the increased number of attackers. These attackers are shown in the Fig 3 as a square block with red color.

Note that all nodes in these areas (extended secure area and restricted allocated sextant) known as candidate nodes have to respond to the routing initiation mechanism (RTS packet) initiated by the source node. The candidate nodes have to reply with with CTS packet but with different responding or replying time in order to prevent attackers from restricted area to reply, capture the communication and drop the packets. Therefore, the proposed protocol explores the modified CTS response time, which is based on the ratio of the distance to the destination residual energy and the priority value related to its geographic position in the greedy forwarding area (higher prioritized node having a shorter response timer). Since geographic routing protocols mostly rely on the geographic forwarding strategy to forwarding data packets, it is assumed that every sensor node in the network is equipped with GPS or localization protocol that defines the node position. Moreover, it assumes that the source node has the knowledge of the location of their destination which can be obtained using location service protocol. The protocol considers the wireless radio links of the sensor nodes are unprotected; if an attacker is able to interact with the network functionalities, it can capture and drop data packets. It is also assumed that the attackers possess hardware capabilities similar to that of legitimate nodes, and wireless transmissions use the same power levels.

### Detail Overview of Proposed Protocol

In this section, a Region-Based Secure Geographic Routing Protocol (SBRGR) protocol that is an improvement of the DWSIGF [20] is described in detail together with the pseudocode of its algorithm. First, the shortcomings of the DWSIGF are presented. DWSIGF employs a dynamic collection window which tolerates and allows a handful of nodes within the restricted allocated sextant to respond with CTS packet to form a candidate set. DWSIGF provides a secure environment in the process of selecting a next-hop node since the attackers in the forwarding area have no knowledge of the precise period of time when the collection window is opened. DWSIGF adopted the ‘*restricted*’ allocated forwarding area in [19, 36]. This area is used since it provides some assurance that CTS responders can overhear each other and terminate their CTS Response timers accordingly. However, when multiple neighboring attackers are in the communication link, this area may provide inadequate CTS responders from which to select a legitimate node. Although there exist appropriate candidate nodes in the shaded area (for example node E and D) as shown in the Fig 2, the sender has to re-initiate communication through retransmission of the control packet with its neighbors when the attacker is selected and drop the data packets. This may not provide a guarantee to the neighbors to successfully receive the data packets again next time due to the error-prone of wireless links which may lead to poor network performance. Also, dynamically opening and closing of the collection window provides insufficient time for the candidate *(competing)* nodes to respond with the CTS packet and be among the contenders for the selection as a relay node. The protocol also utilizes the simultaneous verification to identify the duplicate location of the nodes. However, an attacker is still being selected as a relay node under priority selection criteria.

Therefore, in order to address the aforementioned shortcomings of DWSIGF described above, firstly a secure extended area is introduced. The restricted allocated sextant *(FrwdAreaSector)* in [20] is extended to secure extended area *(SecExtFrwdArea)* so as to admit more nodes in the communication process. This area is introduced to increase forwarding opportunities of the other legitimate nodes rather than those resided in the restricted allocated sextant to reply with CTS packet. The extended area is defined as an area between the transmission arc centered at the distance from the destination to the sender [37] subtracting the area of the sector created by allocated sextant and the transmission radius of the sender [34]. And mathematically, this area can be derived as follows:

Firstly, the area of Sector XYZ is derived as:

$$\begin{array}{c} XYZ=\frac{r^{2}\beta \pi }{360} \end{array}$$ (1)

Where *β* is the Sextant connecting the Sender with the straight line toward the Destination *D* to the point *Y* and *Z* and *r* is the transmission range of the Sender *S*.

Then, the area XVOW between two overlapping circles is obtained as:

$$\begin{array}{c} XVOW=\pi \left(R^{2}-r^{2}\right) \end{array}$$ (2)

Where *R* is radius of the distance from Destination D to Sender S and *R*^2^, *r*^2^ > 0, *R* ≠ *r* and *R* > *r*

Finally, the secure extended area VZXWY is obtained by subtracting Eq (1) from Eq (2) as

$$\begin{array}{c} =\pi \left(R^{2}-r^{2}\right)-\frac{r^{2}\beta \pi }{360} \end{array}$$ (3)

Thus,

$$\begin{array}{c} VZXWY=r^{2}\pi \left(R^{2}-1-\frac{\beta }{360}\right) \end{array}$$ (4)

The proposed secure extended area *(SecExtFrwdArea)* encourages more nodes to participate in forwarding process, which increases the possibility to select a relay node among the increased number of legitimate nodes within the greedy forwarding area *ECABG*. The nodes that reside in the secure extended area have high priority of being selected as a relay node compared to other nodes located in the restricted allocated sextant. These nodes (i.e. from secure extended area) immediately reply with a CTS packet to form a candidate set *(Candidate_List(S2))* in fully distributed way after receiving ORTS packet from the sender. On the other hand, nodes that reside in the allocated sextant have low priority and have to wait for two or more nodes (based on the bound collection window time explained later in this Section) in the secure extended area to reply with CTS packets and before replying with their own CTSs. This strategic halts or at least minimizes CTS responses from nodes with low priority allocated in the given restricted sextant, which may contain the combination of legitimate nodes and the number of attackers which ultimately form a candidate set *(Candidate_List(S1))*. These two sets of candidate nodes from both areas are then combined to form a new candidate set *(Candidate_List(S))* contains a list of candidate nodes that have to pass through a verification process to be explained later in this Section.

To build the candidate set *(Candidate_List(S))*, the proposed protocol modifies a function of CTS response time for forwarding nodes in each region of the greedy forwarding area to engage in the communication process. In order to compute this function, each neighbouring node (closer to the destination than the sender and near to the secure extended area) in the greedy forwarding area sets its own CTS response local timer when it received a broadcast message from the sender to initiate the routing communication. This function is used to enhance the route decision process and is computed as follows: This function is computed as follows:

$$\begin{array}{c} f\left(CTS_{RespT}\right)=\left(1-\frac{D+W+R_{d}}{W_{D}+W_{E}+W_{R}}\right)+\left(1-\frac{\theta }{\theta^{\prime }}\right) \end{array}$$ (5)

Where *D* is measuring the increase distance towards the destination that a packet will take if the node is appropriated the responsibility of transmission. While *W* is measuring the node’s residual energy as a fraction of the maximum energy provided upon deployment. Short CTS response local timer contributes to less parameter D towards the destination i.e. closer node from the destination having a shorter CTS response timer). In case of W, less CTS response timer also contributes to high value of nodes’ residual energy. This makes neighboring nodes with more available energy more likely to take part in the packet transmission. To evenly disperse the system workload, randomization *R* is included in the CTS response local timer. *W*_*D*_, *W*_*E*_, *W*_*R*_ are the weights that can be used to tune the values of distance, energy, and random parameters, respectively based on their importance to the packet transmission process and (*W*_*D*_ + *W*_*E*_ + *W*_*R*_ should be 0).

Finally, *θ* is the sextant from the line connecting the sender S and the destination D to the current node (neighbouring node) *A* ∈ *N*^(*S*)^ and *θ*′ is the sextant from the sender and the destination to the maximum sector sextant as shown in Fig 3. A term $\left(1-\frac{\theta }{\theta^{\prime }}\right)$ represents the increase sextant towards the secure extended area in which the lower the term the closeness of the node towards secure extended area. Namely, the closest node in this area from the sender to the destination has the shortest time. In the greedy routing mode, the value of *θ* and *θ*′ should lie and vary between −90 to 90 and *θ*′ > *θ*

Then, the function of delay priority for each node is computed as follows:

$$\begin{array}{c} f^{\prime }\left(CTS_{RespT}\right)=f\left(CTS_{RespT}\right) \end{array}$$ (6)

If the node says *D* or *E* is in the secure extended area shown in Fig 2, then the function *f’* returns zero; otherwise, it returns *T*_*initial*_ value for other nodes reside in the restricted allocated area (i.e. node *B*). The value of *T*_*initial*_ for each node is given by the application during the network initializing stage and is set to be in the range of 0 ≥ *T*_*initial*_ ≤ *t*_1_, where *t*_1_ is a lower bound collection window interval describes later in this Section. Generally, the nodes in the secure extended area wake up first and rush to reply with CTS packets to form the new candidate set *(Candidate_List(S))*.

Finally, adding Eqs (5) and (6), the function of CTS Local Response Timer for each node is obtained as follows

$$\begin{array}{c} funtion\left(CTSResponseTime\right)=f\left(CTS_{RespT}\right)+f^{\prime }\left(CTS_{RespT}\right), \end{array}$$ (7)

Secondly, SRBGR introduces a bound collection window time that allows the Sender node to tolerate and collect more candidate nodes during the selection process of the relay nodes. The bound collection window consists of *’bound intervals’* that guarantee the lower *t*_1_ and upper *t*_2_ bound that allow more nodes to respond with CTS packet. The lower bound window is defined as the collection time in which at least two forwarding nodes are selected as competing nodes while the upper bound window is a collection time in which competing nodes from seven and above are selected to build a candidate set. These bound intervals were obtained after thorough investigation of the ‘picking pattern’ of candidate nodes (neighbouring nodes) generated by the dynamic collection window of the DWSIGF [20] in which the maximum and minimum number of candidate nodes are selected during the process of building the candidate set. The time to collect the minimum number of candidate nodes corresponds to the lower bound interval of the selection, while the time to collect the maximum number of candidates belongs to the upper bound interval of the selection. The proposed bound window interval (*CW*) is computed as follows:

$$\begin{array}{c} CW=t{}_{1}+rand\left(\right)*(t{}_{2}-t{}_{1}) \end{array}$$ (8)

Where multiplication scales (*) the result of bound window interval from the (0, 1) opens from (0, *t*_2_ − *t*_1_), while the addition (+) translates it by *t*_1_, transforming it to the desired (*t*_2_ − *t*_1_) open interval. Thus, the proposed bound collection window allows more nodes to be collected by the Sender (i.e. first, legitimate nodes from secure extended area and then, if collection window time allows nodes from restricted allocated sextant). This increases the number of legitimate nodes in the selection process, resulting to decreasing of the possibility of attacker selection as a relay node. Consequently, it guarantees high packets deliver to the destination while minimizing retransmission of both control and/or data packets. The value of collection window should be in range of *t*1 ≥ *CW* ≤ *t*_2_.

Finally, verification cost (*VerfCost*) is designed to provide a validation of node location in order to identify and avoid attacker selection when the priority selection criteria is of concern. The *VerfCost* balances the nodes that have desire to forward packets to the destination (i.e. closer to the destination) and the nodes that delay (i.e. not close to the destination) to respond with CTS packet. It is derived as follows: First, the fraction of progress distance (*DistProg*) of the node to the destination is derived as:

$$\begin{array}{c} DistProg=\;\frac{Dist_{S-D}-Dist_{A-D}}{Dist_{S-D}},ifDist_{S-D}>0 \end{array}$$ (9)

Where, *Dist*_*S* − *D*_ is a distance from the Sender *S* to Destination *D* and *Dist*_*A* − *D*_ is a distance from a neighbouring node *A* to the Destination *D*.

Then, the fraction of CTS response time (*CTSTimeW*) of the nodes before responding with CTS packet is computed as:

$$\begin{array}{c} CTSTimeW=\;\frac{CTSRespTime_{A}}{CTSRespTime_{T}},ifCTSRespTime_{T}>0 \end{array}$$ (10)

Where, *CTSRespTime*_*A*_ is the CTS Response time of the neighbouring node *A* and the *CTSRespTime*_*T*_ is the total CTS Response time of the neighbouring nodes.

Finally, adding Eqs (9) and (10) to obtain *VerfCost* as follows:

$$\begin{array}{c} VerfCost=DistProg+CTSTimeW \end{array}$$ (11)

To select a relay node to send packets, the node that minimizes *VerfCost* is selected to forward packet to the destination. The verification cost properly identifies a legitimate relay node and isolate the attacker. The pseudocode for the proposed Secure Region-Based Geographic Routing Protocol is shown in Algorithm 1.

### Complex Analysis for the Proposed Protocol

**Theorem One**: The communication overhead complexity of control packets in the network is *O*(*n*), where *n* is the number of nodes in the network.

**Proof**: To compute the communication overheads in SRBGR let’s assume that at the beginning of the communication process, there are *n* − 1 nodes within the communication range of the source node. Therefore, *n* − *i* (an ORTS packet) from the source node is broadcasted to the candidate nodes (competing nodes) within the communication range. After receiving the broadcasted message, each competing node in response replies with *a* − *i* (a CTS packet) as well as an *a* ACK packet from the selected contender to the sending nodes. Hence, the total number of control packets in the network is (*n* − *i*) + (*a* − *i*) + *a* = *n* + 2*a* − 2*i*. Thus, the communication overhead of the protocol in the network is *O*(*n*).

**Theorem Two**: Predictability and collection response of candidate nodes is maintained.

**Proof**: A dynamic (random) length of the collection window ensures unpredictability with no guarantee for collection of responses. SBBGR introduces a based bound collection window that exhibits dynamism however, within a fixed maximum and minimum bound collection window intervals that guarantee the selection with high predictability and response collection. The predictability and response collection have been further enhanced by the introduction of extended secure area which increases the number of response within a greedy forwarding area.

## Performance Evaluations

This section evaluates the performance of IGF, SIGF, DWSIGF protocols and the proposed SRBGR protocol using simulation developed via MATLAB 7.0. The protocols are evaluated in terms of packet delivery ratio, message overhead, end to end delay and possibility of attacker selection into two scenarios: Attack-Free Communication and Attack-Based Communication. The simulation parameters used are shown in Table 1. The simulation experiments are limited to constant bit rate (CBR) stream of 100 packets with a payload of 32 bytes and radio bandwidth of 200 *kbps*. The experiments use many to many constant bit rate (CBR) flows, which is the multiplication of point-to-point communication expected in such systems, for example, from an event of interest back to a base station.

**Table 1:** Simulation Parameters.

Parameters	Descriptions
*Terrain*	150 × 150 meters
*NumberofNodes*	196
*NodePlacement*	*Grid* + *Gaussiandistribution* = *Grid* + *η*(0, 16)*noise*
*Application*	CBR Flow Streams
*Playload*	32 Bytes
*SimulationLength* *RadioRange*	100 packets, 100 runs 40 Meters
*RadioBandwidth*	200 kb/sec

**Fig 4:**
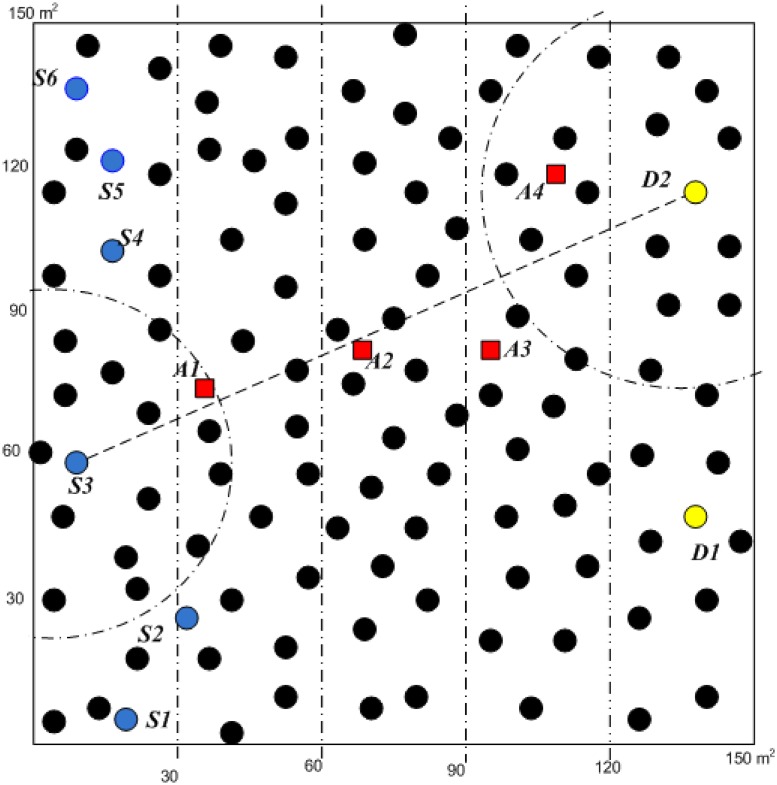
Simulation Terrain Consists of Sensor Nodes, Senders, Destinations and Attackers (A1–A4).

Fig 4 shows a terrain under simulation is 150 × 150 square meters with 196 nodes having the transmission range of 40 meters. The terrain is uniformly divided into 196 cells and each node is placed at the center of the cell and then distributed using a Gaussian distribution with standard deviation of four meters. Then, six Senders and two Destinations are randomly located on the left and right hand sides of the simulation terrain, respectively.

The Figure also illustrates the configuration of attackers’ (A1∧A2∧A3∧A4) positions in the terrain connecting the Sender and the Destination. A1 and A4 are configured to be an optimal relay node in which A1 is located near the sender closer to the routing path while A4 is near the destination and a little bit far from the routing path. The attacker A2 and A3 are classified as non-optimal relays with respect to the sender and the destination, but A2 is near the routing path while A3 is far from the routing path. The reason for setting different attackers’ locations is to study their impact with respect to Sybil and Black hole attacks.

**Algorithm 1** Proposed SRBGR.

**Input**: *SenderS*, *DestinationD*, *NeighbouringNode*(*S*), *RadiusR* **Output**: A Relay Node

**begin**

 $Distance_{S-D}=\sqrt{{\left(X_{S}-X_{D}\right)}^{2}+{\left(Y_{S}-Y_{D}\right)}^{2}}$

 **for** *every node neighbour node CTSresponse* ∈ *Forwarding*_*List*(*S*) **do**

  $Distance_{S-A}=\sqrt{{\left(X_{S}-X_{A}\right)}^{2}+{\left(Y_{S}-Y_{A}\right)}^{2}}$

  $Sextant_{S-A}=cos^{-1}\frac{SK^{2}+SD^{2}+KD^{2}}{2SA*SD}$

  **if** *Distance*_*S* − *A*_ ≤ *Radius*(*S*) and *Sextant*_*S* − *A*_ ≤ *FrwdAreaSector* **then**

   *Candidate*_*List*(*S*1) ← *Candidate*_*List*(*S*) ∪ {*CTSresponse*}

  **if** *Distance*_*S* − *E*_ ≤ *Radius*(*S*) and *Sextant*_*S* − *E*_ ≤ *SecExtFrwdArea* **then**

   *Candidate*_*List*(*S*2) ← *Candidate*_*List*(*S*) ∪ {*CTSresponse*}

 **end**

 *CandidateSet*(*S*) = *Candidate*_*List*(*S*1) ∪ *Candidate*_*List*(*S*2)

 $Distance_{K-D}=\sqrt{{\left(X_{K}-X_{D}\right)}^{2}+{\left(Y_{K}-Y_{D}\right)}^{2}}$

 $f\left(CTS_{RespT}\right)=(1-\;\frac{D+W+R_{d})}{W_{D}+W_{E}+W_{R}}+\left(1-\frac{\theta }{\theta^{\prime }}\right)$

 **for** *every node in Candidate Set* (*S*) **do**

  **if** *CTSresponse* ∈ *SecExtFrwdArea* **then**

   *f*′(*CTS*_*RespT*_) ← ⌀

   else

   *f*′(*CTS*_*RespT*_) = *f*(*T*_*initial*_)

 **end**

 *for each node in the candidate set (S)*

 (*CTSResponseTime*) = *f*(*CTS*_*RespT*_ + *f*′(*CTS*_*RespT*_)

 generate CW //*GenerateCollectionWindow*

 *CW* = *t*_1_ + *rand*() * (*t*_2_ − *t*_1_) //*CollectionWindowboundintervalCW*(*t*1, *t*2)

 **while** (*CW*_*t*1, *t*2 <> ⌀) *and CTSRespTime*(*K*) ← ⌀ **do**

  **if** *CTSResponseReceive and CTS_location* ∈ *FrwdAreasector* **then**

   *Candidate*_*List*(*S*) = *CTSResponse* // collect CTS resposes

 **end**

 **for** *every candidates node CTS response in Candidate_List* (*S*) **do**

  $DistProg=\;\frac{Dist_{S-D}-Dist_{A-D}}{Dist_{S-D}}$

  $CTSTimeW=\;\frac{CTSRespTime_{A}}{CTSRespTime_{T}}$

  *VerfCost* = *DistProg* + *CTSTimeW*

  **if** *Weight*(*CTSResponse*) ≤ *VerfCost*(*CTSResponses*) **then**

   *RelayNode* ← *CTSresponse*

 **end**

 **if** *VerfCost*((*CTSResponse*) ≥ 0.0) **then**

  return *RelayNode*

 **else**

  return *NULL*

 **end**

**end**

### Attack-Free Communication

In this scenario, the experiments are carried out under increasing traffic loads until traffic reaches 12 packets per second. In the experiment, the packet delivery ratio, end to end delay as well as communication (message) overheads are measured. These experiments act as a baseline for comparison when attack-based communication (e.i. Sybil and black hole attacks) is involved. To avoid confusion the term SIGF0 is referred as simply SIGF.

The experiment results demonstrate that under increasing traffic loads, although SRBGR modestly increases end to end delay as well as communication overheads from high number of message exchanges, but it maintains high packet delivery ratio.

**Fig 5:**
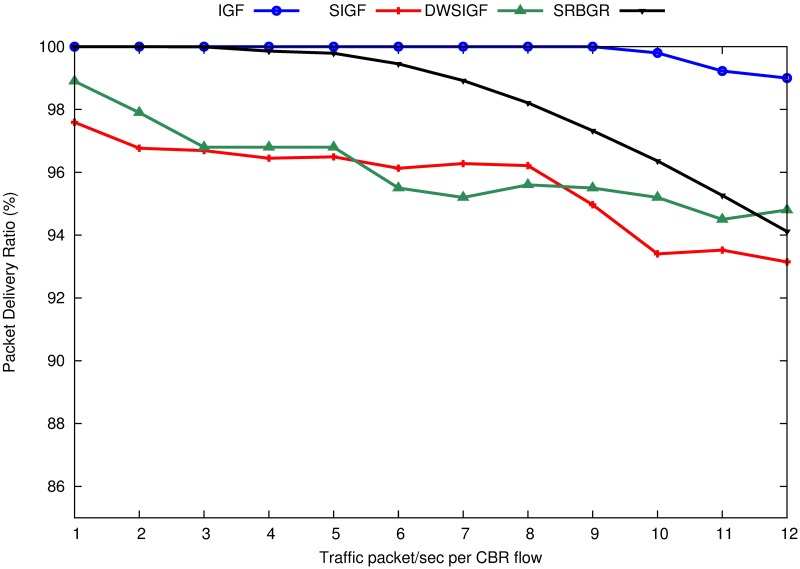
Packet Delivery Ratio (PDR).

Fig 5 shows the packet delivery ratio of IGF, SIGF, DWSIGF and proposed SRBGR protocols. The graphs in the Figure indicate that the SRBGR maintains PDR of 3% and 2.1% higher than SIGF (95%) and DWSIGF (96%), respectively, and 1.6% lower with respect to IGF (99.8%). All protocols with the exception of IGF suffer congestion when traffic flow rates increase to more than 7 packets/second per CBR flow due to high number of collected CTS packets for each ORTS packet. However, SRBGR performs better since it diminishes the failure in routing initiation due to the enough number of nodes to take part in communication.

**Fig 6:**
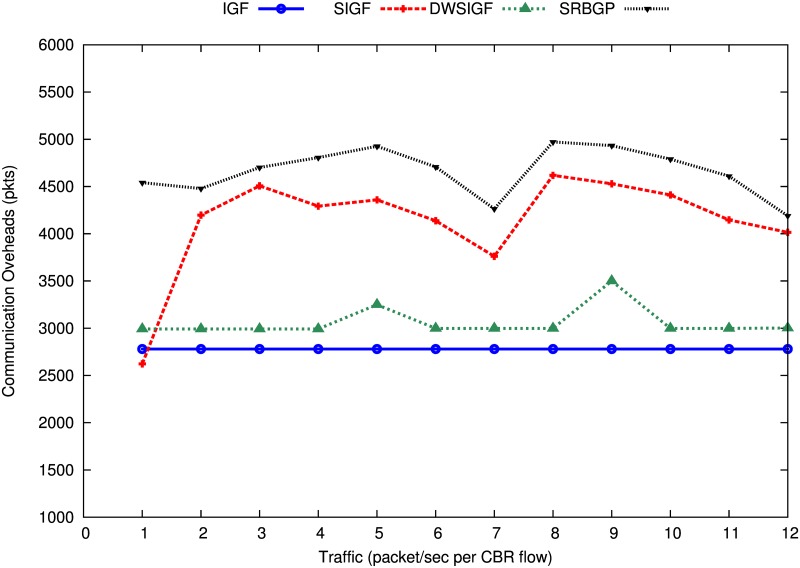
Communication Overheads (packets).

Fig 6 shows communication overheads of the IGF, SIGF, DWSIGF and proposed SRBGR protocols. The Figure illustrates that the SRBGR increases communication overheads compared to the other protocols because it encourages more responders due to the extension of the forwarding area as well as the bound collection window. These responders increase control packets in the communication processes which include ORTS, CTS and ACK for acknowledgment of data packets. The graphs in the Figure indicate that SRBGR maintains an average increment of 40%, 11% and 34% in communication overheads with respective to IGF, SIGF and DWSIGF, respectively.

**Fig 7:**
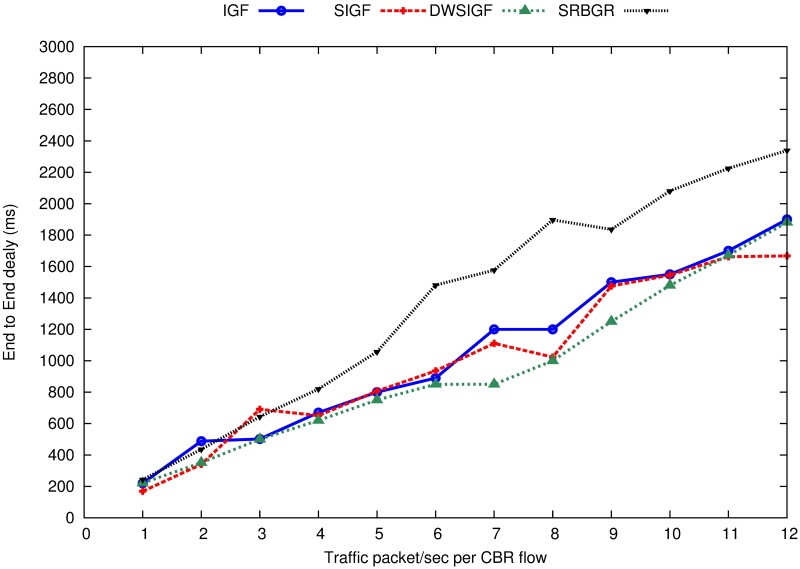
End-to-End Delay (ms).

Fig 7 shows the end-to-end delay of the IGF, SIGF DWSIGF and proposed SRBGR protocols. The Figure illustrates that the SRBGR incurs moderate higher end-to-end delay compared to other protocols when the traffic loads increase. This is due to the high number of hops taken to transmit data packets influenced by the extension of the forwarding area as well as waiting time observed by a Sender of ORTS and additional waiting time used when selecting a relay node. The graphs indicate that SRBGR gradually increases in delay from approximately 488 ms from the beginning of the simulation to the end at 2227 ms.

The results show that the proposed SRBGR adds extra communication overheads and minimum delay, maintains a high packet delivery ratio compared to SIGF and DWSIGF.

### Attack-Based Communication-Sybil Attacks

In the Sybil attacks, the experiments to evaluate SIFG, DWSIGF and proposed SRBGR are carried out under virtual Sybil nodes created by A1 and A2 in two different scenarios. First, attacker A1 and A2 separately create six virtual Sybil nodes randomly located about itself with a Sybil distribution radius (i.e. radio transmission range) under increasing traffic loads. Second, A1 and A2 are involved in creating virtual Sybil nodes and the protocols are investigated under an increasing number os Sybil nodes. In both cases, virtual Sybil nodes are given different identities starting from 197 because the identities lower than these numbers belong to legitimate nodes. Each Sybil node drops all packets (i.e. perform black hole attack) when it’s selected as a relay node. Sybil attack happens when the adversary fabricates new identification or steals identity from lawful nodes as discussed in [33, 38, 39].

#### Under Increase Traffic Load

**Fig 8:**
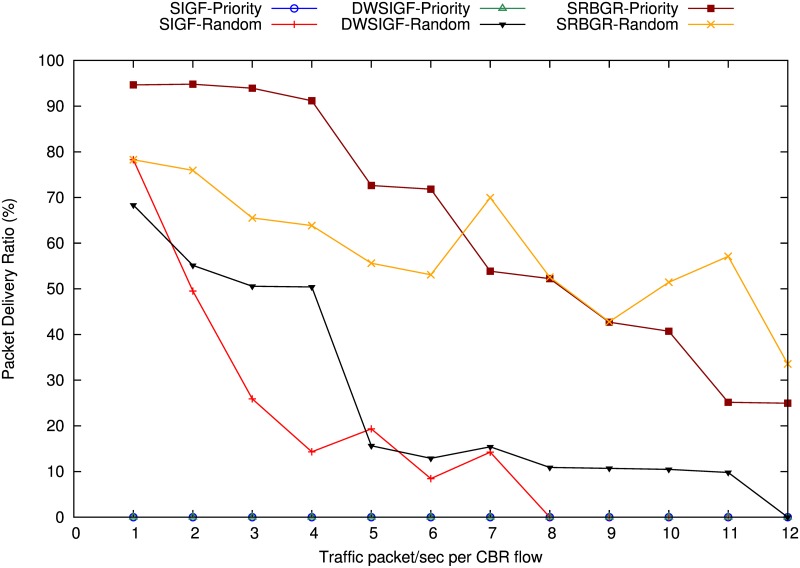
Impact on PDR of A1 under increasing Traffic loads.

**Fig 9:**
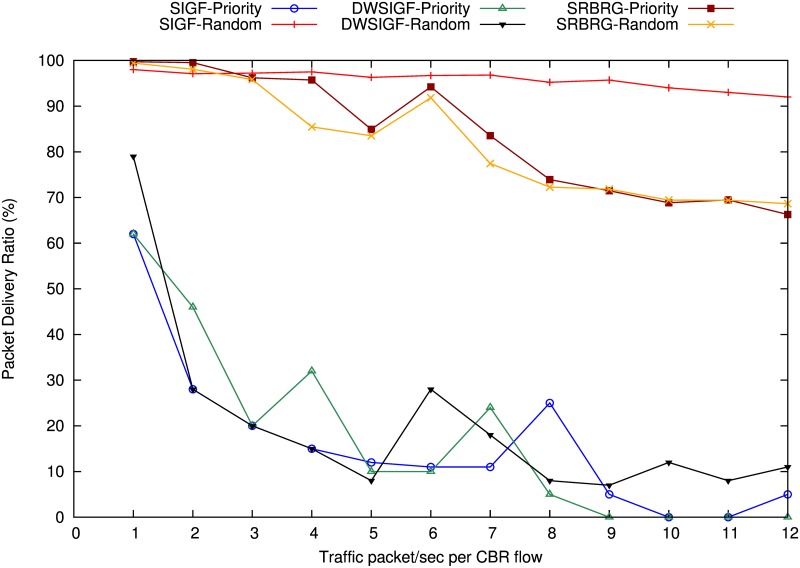
Impact on PDR of A2 under increasing Traffic loads.

Figs 8 and 9 show the experimental results of proposed SRBGR against existing secure routing protocols under Sybil attack by A1 and A2 in different traffic loads.

A1: In the Fig 8 SRBGR performs better compared to SIGF and DWSIGF. SRBGR priority maintains higher PDR of (63%) compared to SIGF priority (0%) and DWSIGF priority (0%) which always select A1 as relay node. The existence of legitimate nodes from secure extended area in SRBGR allows these nodes to participate in the routing process while the process of verification manages to identify and isolate attackers when it maximizes verification cost; hence, improves packet delivery to the destination. SRBGR random has an increment of 40% and 32% in PDR compared to SIGF(18%) and DWSIGF (26%). SRBGR random experiences relative spike in PDR due to the stable routes after coming from moderate PDR degradation in seven and eleven traffic flow rates. The existence of more legitimate nodes in secure extended area and their priorities in responding to ORTS message minimizes the chance of selecting an attacker, consequently, it improves the packet delivery ratio in SRBGR random.

A2: In Fig 9 the proposed SRBGR outperforms SIGF and DWSIGF protocols in the PDR under light traffic load. SRBGR priority performs better with PDR of 83% compared to the DWSIGF priority with 20% PDR. This is because the A2 is not an optimal relay node and even if an attacker manages to reply with CTS packet, SRBGR identifies and eliminates the attacker due to its higher verification cost. SRBGR random also is robust against the attacker and achieves an average of 81% in PDR higher compared to SIGF 16% and DWSIGF 17%. The existence of more legitimate nodes, competing for the selection to become a relay node influenced by the extension of secured extended area, improves packet delivery to the destination for SRBGR random.

#### Increase Number of Sybil Nodes

**Fig 10:**
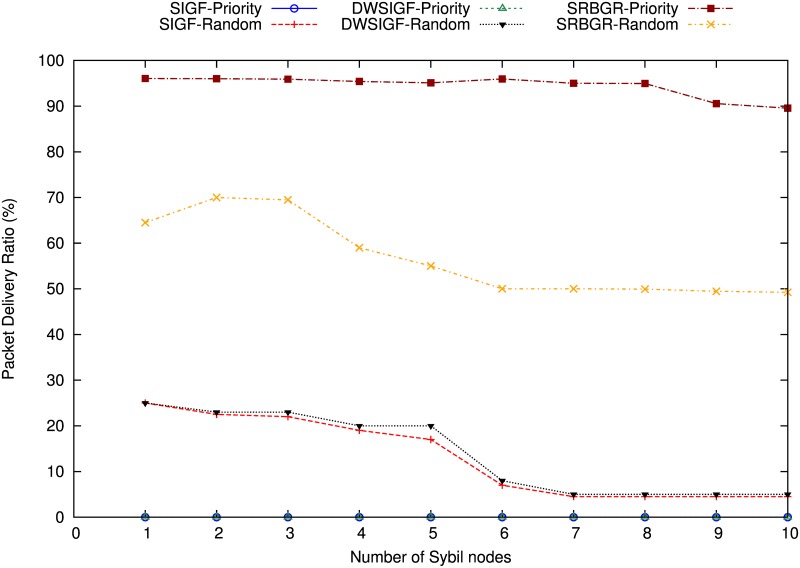
Impact on PDR of A1 Under Increasing Number of Sybil virtual nodes.

**Fig 11:**
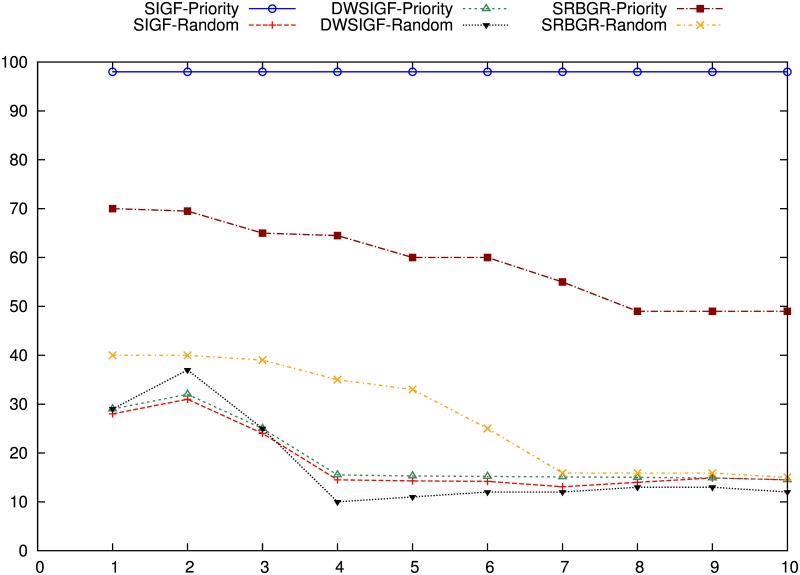
Impact on PDR of A2 Under Increasing Number of Sybil virtual nodes.

Figs 10 and 11 show the experimental results of proposed SRBGR against other secure routing protocols under increasing Sybil attack (i.e. 12 virtual nodes) created by A1 and A2.

A1: The Fig 10 demonstrates that despite an increase in number of Sybil virtual nodes, SRBGR priority achieves a high packet delivery ratio compared to other protocols when A1 is involved. SRBGR maintains PDR of 95% throughout (i.e. increase number of virtual nodes) and moderately decline to 90% when the number of Sybil nodes is greater than 8. The better performance of SRBGR is due to both secure extended area and verification cost which encourage more legitimate nodes and isolation of the attacker respectively. Consequently, it improves packet delivery to the destination compared to the other protocols. In randomized protocols for SRBGR, SIGF and DWSIGF fare worse, however, the later still outperforms others and achieves 60% PDR higher compared to 30% and 25% of SIGF and DWSIGF respectively.

A2: The Fig 11 demonstrates that overall PDR declines considerably when the number of Sybil nodes increases. However, SIGF and SRBGR priority performed better whereas the later achieves almost an average of 95% PDR. SRBGR priority outperforms DWSIGF in PDR with an average PDR of 58% compared to 15% of DWSIGF. Even in the existence of virtual Sybil nodes SRBGR identifies attacker using verification cost and hence improves PDR. In randomized protocols, SRBGR shows better performance with 28% PDR compared to SIGF and DWSIGF with 18% and 17% respectively. The response of legitimate nodes from the secure extended area to participate in communication process increases the probability of their selection and isolates the attackers.

### Attack-based Communication -Black hole

In the black hole attacks, the experiments are carried out when the attackers are in the communication link under seven packets per second of traffic load flow in order to avoid network congestion. A black hole attack is created when the attacker receives a stream of packets from legitimate nodes and drops all packets as discussed in [39]. In the experiments, two cases were considered to help execute black hole: the attack with and without the help of CTS rushing attack [40]. CTS rushing ignores all routing disciplines and rushes to become the first one to respond to CTS packet so that it can be received first by the ORTS Sender. When the attacker is selected as a relay node, it just drops packets without forwarding to the next hop or to the destination. The attacks were performed by each single attacker (A1, A2, A3 and A4) and multiple attackers (A1∧A2∧A3∧A4) located at different positions in the simulation terrain as shown in Fig 4.

#### Without CTS Rushing Attacks

**Fig 12:**
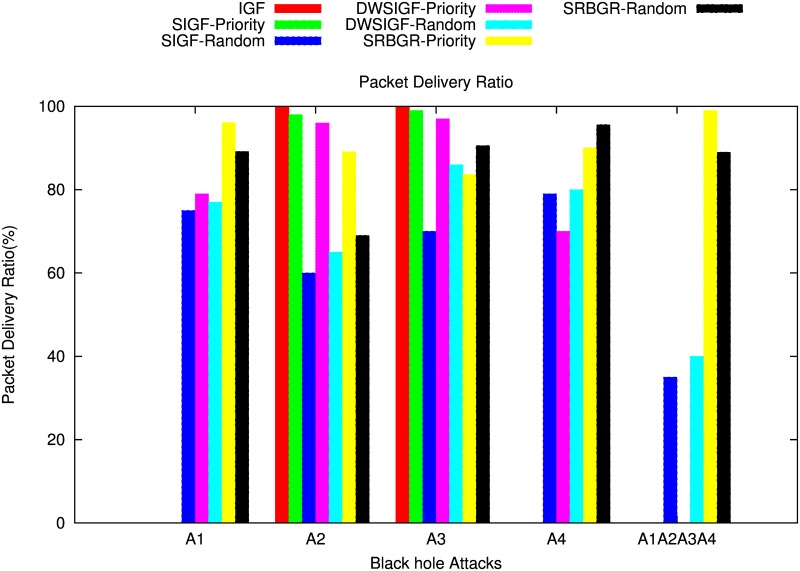
Packet Delivery Ratio: The impack of Blackhole Attackers (A1, A2, A3, A4, A1∧A2∧A3∧A4).

Fig 12 shows the impact of the black hole attack on PDR of SIGF, DWSIGF as well as the proposed SRBGR protocols. The figure demonstrates that SRBGR is robust against the involvement of A1 compared to IGF, SIGF and DWSIGF. SRBGR priority maintains 95%, 95% and 28% PDR better than IGF (0%), SIGF priority (0%) and DWSIGF priority (67%), respectively. This is because the secure extended area proposed produces legitimate nodes with high priority of being selected as relay nodes since they reply first with CTS (i.e. CTS response time expires first) to form a candidate set for the selection. Even if the A1 replied with CTS, it might not be selected as a relay node since it would have maximized verification cost.

**Fig 13:**
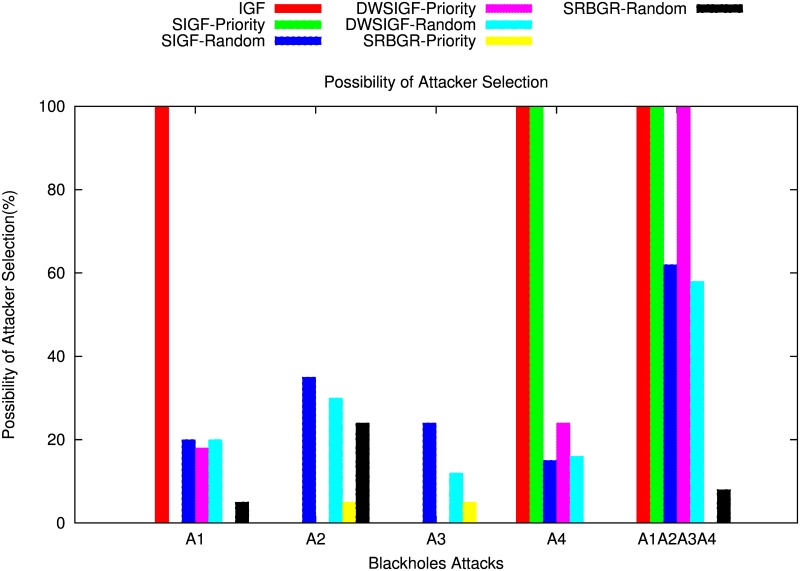
Possibility of Attacker Selection.

On the other hand, in randomized protocols, SRBGR have PDR increments of 14% and 12% higher compared to SIGF(75%) and DWSIGF(77%), respectively. This is because the bound collection window and secure extended area provide enough time and encourages more legitimate nodes to participate in the communication process and thus increases chances of their selection as an appropriate relay node. Fig 13 also shows the possibility of attacker selection is minimized for SRBGR in both random and priority selection.

When A2 is in the communication link, IGF, SIGF and DWSIGF priority-based have better performance compared to the SRBGR priority as shown in Fig 12. The former protocols achieve PDR of 100%, 98% and 96%, respectively higher than the SRBGR priority with 90%. Although A2 is non optimal relay, SRBGR priority moderately declines PDR since the attacker A2 manages to locate its self in secure extended area and replies with a CTS packet to be selected as a relay node. On the other hand, performance of SRBGR random increases the PDR to 9% and 4% with respect to SIGF random (60%) and DWSIGF random (65%) respectively, due to the high number of legitimate nodes from candidates set facilitated by secured extended area.

Fig 12 shows the performance of the protocols when A3 is in communication link. SRBGR priority maintains PDR of 83% relative lower compared to IGF, SIGF and DWSIGF priority-based protocols with 100%, 99%, and 97% respectively. This is because SRBGR may still select A3 as a relay node since it manages to reply with a CTS packet. On the other hand, SRBGR random maintains good PDR with increments of 21% and 5% with respect to SIGF (70%) and DWSIGF (86%), respectively. This is because the secure extended area used as well as bound collection window time allocated to collect forwarding nodes allows more legitimate nodes’ responses to form a candidate set which facilitated the best random selection of an appropriate relay node. The possibility of attacker selection for SRBGR priority in this case is moderately decreased as shown in Fig 13

Fig 12 demonstrates that the performance of the proposed SRBGR protocol is extremely better, in contrast with IGF, SIGF and DWSIGF even when A4 is an optimal relay. The Figure shows that SRBGR priority achieves 90% PDR higher compared to IGF (0%), SIGF (0%) and DWSIGF (70%). This is because the legitimate nodes located in secure extended area in SRBGR have high priority in replying with CTS packet, and maintain better verification cost hence increase the chance of being selected as an appropriate node. While IGF and SIGF always select A4 and fail to deliver a single packet to the destination.

Also, SRBGR random reduces attacker selection and improves PDR to 11% and 12% better compared to SIGF random (79%) and DWSIGF random (80%). This is because the bound collection window used as well as extended area allocated by the SRBGR provide adequate time and more spaces for more legitimate nodes to participate in the communication. Thus increase chances of their selection while other protocols sometimes allow the selection of A4.

Fig 12 also illustrates the performance of proposed SRBGR and IGF, SIGF and DWSIGF protocols against accumulation of all attackers (A1∧A2∧A3∧A4) in the communication link. In the Figure, it is shown that SRBGR priority outperforms other protocols in terms of PDR. It achieves almost 99% in the PDR while IGF priority, SIGF priority and DWSIGF priority are unable to deliver a single packet to the destination since they always select A1 or the other attackers when they manage to bypass A1. The performance of SRBGR priority is excellent since the extended area allows legitimate nodes to respond quickly with CTS packet since they have minimum CTS respond time (i.e. have high priority) compared to other nodes in the restricted allocated sextant. In addition, when attackers manage to respond with CTS packets, they may fail in the verification process since they have maximum CTS response time which maximizes verification cost as well. In randomized protocols, SIGF and DWSIGF improve PDR compared to its poor results in priority based. However, SRBGR still performs better with 89% PDR compared to 35% and 40% of SIGF and DWSIGF, respectively. In this case, SRBGR reduces attacker selection due to the existence of more legitimate nodes caused by extending the allocated sextant as well as bound collection window which increases the number CTS responses and hence minimizes the possibility of attacker selection. SRBGR minimizes the probability of attacker selection as compared to other protocols as shown in Fig 13.

#### With CTS Rushing Attacks

**Fig 14:**
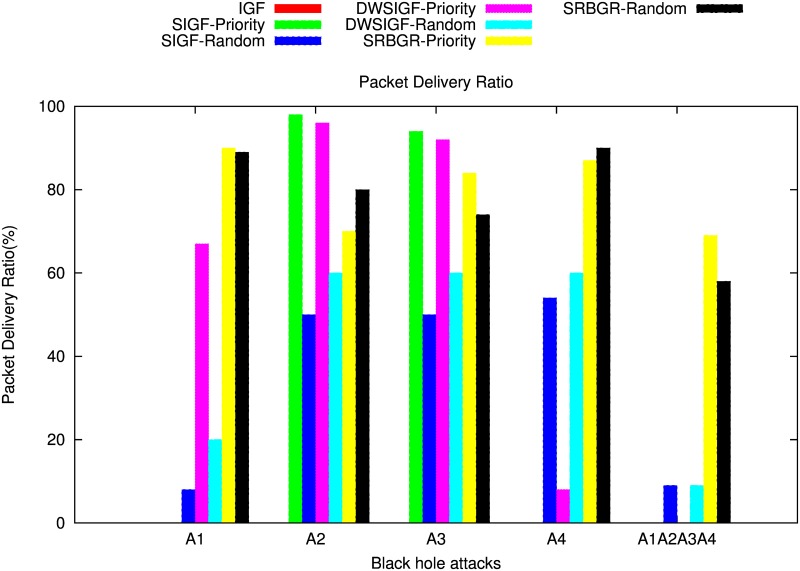
Packet Delivery Ratio: The impack of Blackhole Attackers (A1, A2, A3, A4, A1∧A2∧A3∧A4).

**Fig 15:**
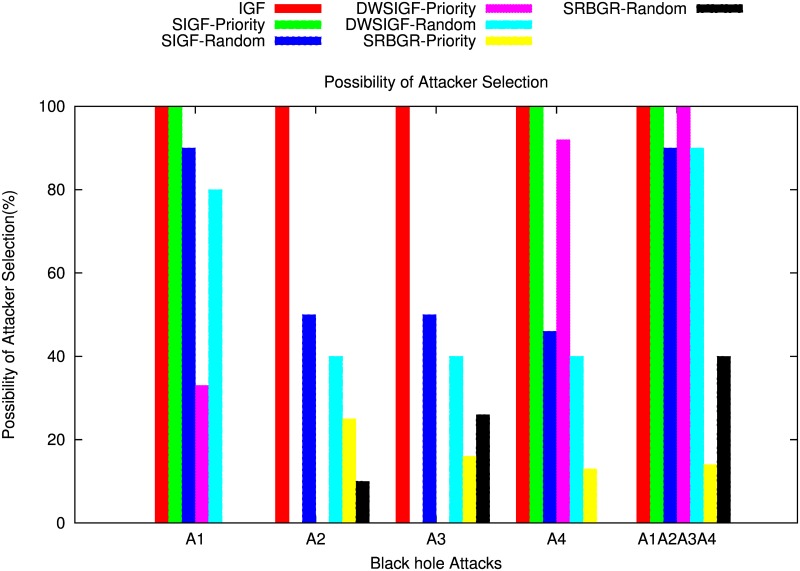
Possibility of Attacker Selection.

Figs 14 and 15 show the impact of the black hole attack on the PDR and the possibility of attacker selection of SIGF, DWSIGF and the proposed SRBGR protocols when attackers are in communication link.

The Fig 14, demonstrates that the SRBGR priority performs better compared to other secure protocols as it completely minimizes the danger of selecting A1 as a relay even if it rushes to be the first to reply with CTS packet. SRBGR achieves almost 90% PDR better than IGF (0%), SIFG priority (0%) and DWSIGF priority (67%). This is because the legitimate nodes in the secure extended area have high priority to respond with CTS packets hence minimizes their verification cost and improves the chance of their selection. In randomized protocols, SRBGR maintains PDR of 89% better than the PDRs of SIGF and DWSIGF with 8% and 20%, respectively. The bound collection window used in SRBGR increases the CTS responses of legitimate nodes from secure extended area, and hence increases their chances of being selected as best relay node while SIGF and DWSIGF allow A1 to take part in communication, thus increases the probability of attacker selection and reduces the PDR.

When A2 is involved with CTS rushing attack, SIGF and DWSIGF priority-based have better performance compared to the SRBGR priority as shown in Fig 14. The former protocols achieve PDR of 98% and 96%, respectively higher than 70% of SRBGR priority since A2 is a non- optimal relay and is not favourable for the selection. However, in SRBGR few attackers were selected and hence drop packets while IGF always select A2 resulting to 0% PDR. On the contrary, SRBGR random improves PDR by 30% and 20% better in contrast to SIGF random (50%) and DWSIGF random (60%), respectively. SIGF and DWSIGF levels off due to inadequate time to allow nodes to engage in communication process contrary to SRBGR which allows more nodes to be involved in the selection process as a result of sufficient time allocated by a bound collection window as well as the extended area which increases the selection of legitimate nodes that successfully send packets to the destination.

Also, in case of A3 involvement with CTS rushing attack, Fig 14 illustrates that the proposed SRBGR generally has the same performance as in A2. SRBGR priority achieves 84% PDR however, 10% and 8% lower than SIGF (94%) and DWSIGF (92%), respectively. IGF is still vulnerable to A3 which drops all packets when selected at all runs. SRBGR random, on the other hand, maintains increments of 24% and 14% in PDR better with respect to SIGF (50%) and DWSIGF (60%). SRBGR random performs well compared to other protocols since it allows more legitimate nodes to participate in communication. Fig 14 illustrates the performance of the IGF, SIGF, DWSIGF and proposed SRBGR protocols when A4 with CTS rushing attack is involved. IGF, SIGF priority and DWSIGF priority-based protocols are vulnerable to A4 and were generally unable to deliver a single packet since the attacker is always selected as relay node. On the other hand, SRBGR priority maintains 87% better compared to 0%, 0% and 8% of the IGF, SIGF and DWSIGF, respectively. SRBGR shows great robustness since the legitimate nodes from secure extended area have high priority and manage to reply with CTS and hence isolate A4 during the verification process. Also, SRBGR random still outperforms other protocols in PDR with 90% compared to 54% and 60% of SIGF and DWSIGF, respectively. Extended area as well as bound collection window implemented in the SRBGR allow more legitimate nodes participation in communication hence improve the packet delivery ratio.

Fig 14 shows the performance in terms of the PDR of IGF, SIGF, DWSIGF and proposed SRBGR protocols when all attackers (A1∧A2∧A3∧A4) are in communication link. In the Figure, SRBGR priority achieves 69% better than other priority-based protocols (IGF, SIGF priority and DWSIGF) with zero PDR and 100% possibility of attacker selection as shown in Fig 15. Even in the presence of multiple attackers and CTS rushing, the proposed SRBGR protocol produces excellent results. This is becasue the secure extended area allows legitimate nodes to quickly respond to CTS packets and increases the chance of their selection and isolate the attacker hence improve packet delivery to the destination. The attackers are identified and isolated since they fail in the verification process by maximizing their verification cost. In the case of randomness, SIGF and DWSIGF again perform poorly with 9% and 5% PDR, respectively since their routing principles allow attacker selection as a relay node. SRBGR shows robustness in providing defense and achieves 58% PDR since the bound collection window as well as extended area are used to increase the number of legitimate nodes, hence reduce the chances of attacker selection.

Therefore, these results show, in a dangerous situation, the techniques implemented by SRBGR provide enough and robust defense against Sybil and Black hole attacks.

## Conclusion

In this paper a Secure Region-Based Geographic routing protocol (SRBGR) Protocol for WSNs is presented to increase the number of legitimate nodes participating in communication process when the number of attackers increase. SRBGR has flexibility of supporting independency on responding messages, extension of forwarding area, bound collection window so as to collect more nodes as well as a verification process to identify and isolate the attackers. These security measures improve the selection of legitimate nodes in the process of routing packets to a destination. The experiment results demonstrate that the proposed protocol has excellent network performance compared to the existing routing protocols in downgrading the possibility of attacker selection and upgrading the packet delivery ratio. A Plan for future work is to investigate the proposed protocol against the impact of increasing number of nodes with different scenarios of network terrain.

## Supporting Information

S1 PlotData This is the S1 PlotData title. (ZIP)
